# Highly Sensitive Dissolved Oxygen Sensor with High Stability in Seawater Based on Silica-Encapsulated Platinum(II) Porphyrin

**DOI:** 10.3390/s25113559

**Published:** 2025-06-05

**Authors:** Hang Lv, Siyuan Cheng, Song Hu, Guohong Zhou

**Affiliations:** 1 State Key Laboratory of High Performance Ceramics and Superfine Microstructure, Shanghai Institute of Ceramics, Chinese Academy of Sciences, Shanghai 200050, Chinayzhusong10@mail.sic.ac.cn (S.H.); 2 Center of Materials Science and Optoelectronics Engineering, University of Chinese Academy of Sciences, Beijing 100049, China

**Keywords:** dissolved oxygen, sensing, silica-encapsulated PtOEP, fluorescence quenching

## Abstract

This study utilized tetramethylammonium hydroxide (TMAH) as a substitute for traditional catalysts and successfully incorporated platinum octaethylporphyrin (PtOEP) into SiO_2_ nanoparticles (PtOEP@SiO_2_) via the Stöber method. Methyl silicone resin was employed as the matrix material, and a drop-coating technique was applied to fabricate thin films of PtOEP@SiO_2_ particles for dissolved oxygen (DO) sensing in seawater. By optimizing the concentrations of TMAH and PtOEP, a highly sensitive oxygen-sensing film with a quenching ratio (I_0_/I_100_) of 28 was ultimately developed, with a wide linear detection range (0~20 mg/L, R^2^ = 0.994). Stability tests revealed no significant performance degradation during five oxygen–nitrogen cycle tests. After 30 days of immersion in East China Sea seawater, the quenching ratio decreased by only 6%, confirming its long-term stability and excellent resistance to ion interference. This research provides a novel strategy for developing highly reliable in situ marine DO sensors.

## 1. Introduction

Molecular oxygen serves as a fundamental substance for supporting life activities on Earth [1]. Real-time monitoring of DO holds significant importance in environmental protection [2], biological fermentation [3], aquaculture [4], and marine exploration [5]. The Winkler method, the earliest DO detection technique, relies on the oxidation reaction between DO and manganese(II) in alkaline solutions to form high-valent manganese oxides, followed by acidification to release free iodine for quantitative determination via sodium thiosulfate titration [6]. This method demonstrates high precision and reliability; however, it involves cumbersome procedures and cannot achieve real-time monitoring [7]. Developed in the mid-20th century, the Clark electrode method determines DO concentration by measuring current signals generated from oxygen reduction reactions at the cathode [8]. However, such a method requires periodic electrolyte replacement and continuously consumes oxygen during operation, resulting in insufficient long-term stability [9]. The recently developed optical method for DO measurement operates on the principle of dynamic fluorescence (or phosphorescence) quenching [10]. It employs oxygen-specific photoluminescent substances as optical probes, where oxygen acts as a quencher. When oxygen molecules collide with excited-state photoluminescent probes, they transfer partial energy, enabling the probes to return directly to their ground state without emitting photons, thereby reducing photoluminescence intensity. As dissolved oxygen concentration changes, the collision probability between oxygen molecules and the probes alters, leading to variations in photoluminescence intensity. By leveraging this property, the DO concentration in water can be inferred. This method overcomes the limitations of traditional DO detection techniques, offering high accuracy, excellent stability, and long-term in situ monitoring capabilities [11]. Consequently, optical DO sensors based on dynamic fluorescence (or phosphorescence) quenching principles have attracted extensive research attention and have promising applications in ecological environment monitoring and marine ranch water quality assessment [12].

Fluorescent sensing films serve as the core components of optical sensors, typically composed of fluorescent probes and a matrix material for probe immobilization. Commonly employed fluorescent probes include ruthenium(II) complexes [13], platinum-based porphyrins such as platinum tetrakis(pentafluorophenyl) porphine (PtTFPP) [14], and PtOEP [15]. Platinum-based porphyrins have gained widespread application as oxygen-sensitive probes due to their superior photophysical properties, including high quantum yield, large Stokes shift, and extended photoluminescence lifetime [16]. Organically modified silicates have been widely adopted as matrix materials in fluorescent sensing films. Their porous architecture facilitates rapid oxygen diffusion while effectively encapsulating and dispersing fluorescent probe molecules, thereby enabling the fabrication of films with enhanced quenching efficiency [17]. In a representative study, Tang et al. [18] developed homogeneous crack-free films via spin-coating, utilizing silicates derived from n-Octyltriethoxysilane (Octyl-triEOS)/tetraethyl orthosilicate (TEOS) as the matrix combined with tris(4,7-diphenyl-1,10-phenanthroline)ruthenium(II) ([Ru(bpy)_3_]^2+^). However, when considering marine applications, the inherent porosity of these silicates allows direct interaction between immobilized fluorescent probes and diverse ionic species in seawater, potentially compromising the photostability and sensing reliability of the probe molecules.

To enhance the performance of fluorescent probes, researchers have explored nanoparticle encapsulation strategies. Zhang et al. [19] developed DO sensors by embedding PtOEP into polystyrene beads through a swelling method, demonstrating successful detection in river water and rainwater. However, polystyrene is prone to swelling and structural degradation, rendering it unsuitable for seawater environments [20]. According to Bhardwaj et al. [21], forming a silica layer on electrode surfaces during seawater electrolysis reduced ion permeability in seawater by three orders of magnitude while maintaining efficient oxygen transport, demonstrating silica’s excellent marine compatibility. SiO_2_ shells effectively block ionic permeation while maintaining oxygen permeability, making them ideal probe-host materials for marine DO detection applications. The Stöber method remains the predominant approach for synthesizing SiO_2_ nanoparticles, involving the hydrolysis and condensation of TEOS in ethanol/water mixtures catalyzed by ammonium hydroxide. Ding et al. [22] incorporated [Ru(bpy)_3_]^2+^ into the Stöber reaction system, successfully synthesizing [Ru(bpy)_3_]^2+^-loaded SiO_2_ nanoparticles for intracellular DO monitoring. The platinum-based porphyrins generally exhibited superior oxygen responsiveness compared to ruthenium complexes. However, the results highlighted a critical limitation of the conventional Stöber method, which is that the encapsulation efficiency for platinum-based porphyrins is insufficient.

In the traditional Stöber method, ammonia is used as the catalyst, resulting in a relatively slow growth rate of SiO_2_, which in turn causes the nanostructure to become compact. Meanwhile, due to the fluorinated and ethyl-substituted groups in commonly used platinum-based porphyrin oxygen-sensitive probes (PtTFPP and PtOEP), these probes exhibit hydrophobicity, making them difficult to embed into SiO_2_ particles. To address this issue, Chu et al. [17] modified the multi-step reaction process by extending reaction time, periodically replenishing TEOS/ammonia to optimize particle growth dynamics, and successfully achieved PtTFPP encapsulation. However, this method remains procedurally cumbersome and requires prolonged reaction times. Han et al. [23] proposed a TMAH-catalyzed Stöber method modification, enabling rapid SiO_2_ nanoparticle growth in the early stages to form a porous internal structure, while slowing growth in later stages to create a dense outer shell. Building on this work, our study employs TMAH as the catalyst for the modified Stöber process, utilizing platinum octaethylporphyrin (PtOEP) as the fluorescent indicator. This strategy ensures PtOEP is embedded into the porous interior during the rapid initial growth phase, facilitating oxygen diffusion within the particles to enhance oxygen response efficiency. Simultaneously, the subsequently formed dense SiO_2_ shell acts as a barrier, effectively isolating PtOEP from external ion interference. Through systematic modulation of TMAH concentration and PtOEP loading ratios, we successfully engineered SiO_2_ nanoparticles encapsulating PtOEP (PtOEP/SiO_2_) with optimized DO responsiveness. These functional nanoparticles were subsequently blended with silicone resin and deposited as thin films via a drop-casting method. The film stability test in seawater revealed that the quenching ratio decreased by only 6% within one month, demonstrating excellent resistance to interference from various ions in the marine environment.

## 2. Materials and Methods

### 2.1. Materials Preparation

1 mL of TEOS (98%, Aladdin, Shanghai, China), 5 mg of PtOEP (95%, Aladdin, Shanghai, China), and 50 mL of anhydrous ethanol (99.7%, Lingfeng Corporation, Shanghai, China) were combined in a three-necked flask and stirred magnetically at 250 rpm for 10 min. Subsequently, 1 mL of deionized water and 1 mL of TMAH aqueous solution (25% stock solution, Aladdin, Shanghai, China) with mass fractions of 1 wt.%, 1.5 wt.%, 2 wt.%, 2.5 wt.% and 3 wt.% were mixed with continuous magnetic stirring at 250 rpm for 3 h to ensure complete hydrolysis of the TEOS, while maintaining the container sealed throughout the reaction. The reaction mixtures were then centrifuged at 8000 rpm for 5 min to isolate PtOEP@SiO_2_ precipitates, which were alternately washed with ethyl acetate (99.7%, Sinopharm Chemical Reagent Co., Ltd., Shanghai, China) and anhydrous ethanol until the supernatant became transparent, colorless, and fluorescence-free. Finally, the precipitates were dried in a forced-air oven at 60 °C for 4 h to obtain different sizes of PtOEP@SiO_2_ powder.

For the preparation of PtOEP@SiO_2_ particles with different PtOEP loadings, 1 mL of TEOS, varying amounts of PtOEP (2.5 mg, 5 mg, 7.5 mg, or 10 mg) and 50 mL of anhydrous ethanol were combined in a three-necked flask and stirred magnetically at 250 rpm for 10 min. Then 1 mL of deionized water and 1 mL of TMAH aqueous solution (2.5 wt.%) were added and thoroughly mixed by magnetic stirring at 250 rpm for 3 h to achieve full hydrolysis of TEOS. The products were washed and dried at 60 °C for 4 h to obtain SiO_2_ particles embedded with different PtOEP concentrations.

The thin film was fabricated using the drop-coating method. First, 200 mg of PtOEP/SiO_2_ particles and 0.5 mL of a 50% methyl silicone resin benzene solution (Jipeng Fluorosilicone Materials Co., Ltd., Shenzhen, China) were ultrasonically mixed for 30 min. Then, 0.5 mL of anhydrous ethanol was added, and the mixture underwent another 30 min of ultrasonic mixing to obtain the coating solution. Then, 15 mm × 15 mm square glass plates, serving as substrates for the thin films, were ultrasonically cleaned with anhydrous ethanol for 5 min. Subsequently, 60 μL of the coating solution was drop-coated onto the glass substrate and cured in an oven at 60 °C for 5 min.

### 2.2. Characterization

**Figure 1:**
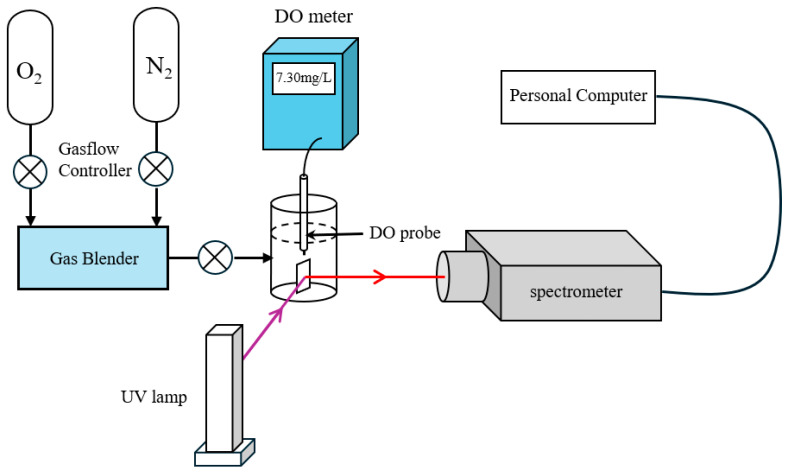
The fluorescence quenching-based DO sensing setup.

The morphologies of the as-prepared nanoparticles were observed by SEM. The DO response capability of the film was tested using a custom-built setup (Figure 1). The chamber for testing DO was a 100 mL quartz glass three-necked flask. During testing, 60 mL of deionized water at 25 °C was added to the flask, and nitrogen or oxygen gas was bubbled into the water at a flow rate of 100 mL/min. When nitrogen gas is introduced into water, the partial pressure of oxygen decreases, thereby reducing the dissolved oxygen concentration; conversely, when oxygen gas is introduced, the partial pressure increases, leading to an elevation in dissolved oxygen concentration. The oxygen partial pressure was controlled by adjusting the gas flow duration to change the DO concentration. Once the concentration stabilized, the reading on the DO meter (JPBJ-610L, Leici Instrument Ltd., Shanghai, China) was recorded. Subsequently, the photoluminescence spectrum of the film was measured using a photoluminescence spectrometer (FLS980, Edinburgh Instruments Ltd., Livingston, UK) at this DO concentration. The ambient temperature was maintained at 25 °C throughout the experiment.

## 3. Results and Discussion

**Figure 2:**
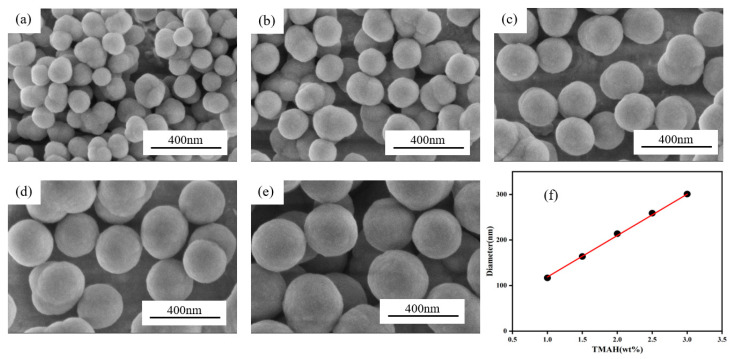
(**a**–**e**) SEM images of silica-encapsulated PtOEP particles with different amounts of TMAH and (**f**) plot of silica-encapsulated PtOEP particle diameter versus TMAH additions.

Figure 2a–e shows the SEM images of silica-encapsulated PtOEP particles prepared using 5 mg PtOEP with different TMAH mass fractions. All of the particles are monodispersed and exhibited spherical characteristics, which would contribute to the uniform photoluminescence of the films. It was statistically measured that the average particle sizes of the silica-encapsulated PtOEP prepared using 1.0, 1.5, 2.0, 2.5, and 3.0 wt.% TMAH were 117 nm, 164 nm, 214 nm, 259 nm, and 301 nm, respectively. The plot of silica-encapsulated PtOEP particle diameter versus TMAH additions shown in Figure 2f indicates that the size of silica-encapsulated PtOEP particles increases linearly with the mass fraction of TMAH, which is consistent with the findings reported by Han et al. [23].

**Figure 3:**
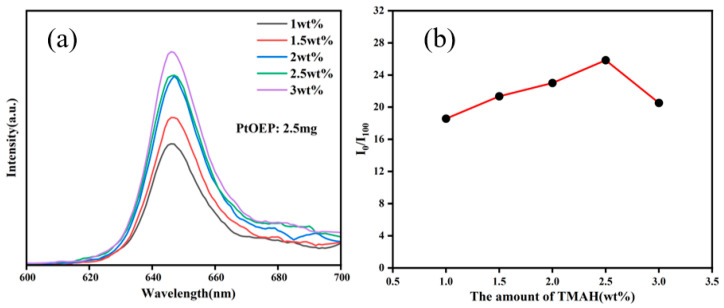
(**a**) Photoluminescence spectra and (**b**) quenching ratio (I_0_/I_100_) of PtOEP/SiO_2_ particles with different TMAH additions.

The photoluminescence emission spectra of PtOEP/SiO_2_ particles prepared with 5 mg PtOEP and varying mass fractions of TMAH are shown in Figure 3a, with an excitation wavelength of 380 nm. It can be seen that photoluminescence intensity enhances steadily with increasing TMAH mass fraction. To evaluate the oxygen sensitivity of different particles, we introduced the concept of the quenching ratio (I_0_/I_100_), defined as the ratio of photoluminescence intensity under zero DO conditions (I_0_) to that under saturated DO conditions (I_100_). A higher I_0_/I_100_ value indicates greater sensitivity. For ease of measurement, the particles were first fabricated into thin films before testing the quenching ratio. The quenching ratios (I_0_/I_100_) of different films verses their TMAH mass fractions were tested and are shown in Figure 3b. The results demonstrate that at lower TMAH mass fractions, as TMAH content increases oxygen sensitivity improves. The optimal oxygen sensitivity is achieved at 2.5 wt.% TMAH, where the I_0_/I_100_ value reaches 28. However, when further increasing the TMAH mass fraction beyond 2.5 wt.% the quenching ratio (I_0_/I_100_) starts to decrease, indicating lower oxygen sensitivity. It was speculated that as the particle size grows, the embedded PtOEP content gradually increases, contributing to the enhancement of oxygen sensitivity. However, with higher TMAH mass fraction, significantly increased particle size would hinder oxygen diffusion within the nanoparticles and consequently reduce the oxygen sensitivity of the film.

**Figure 4:**
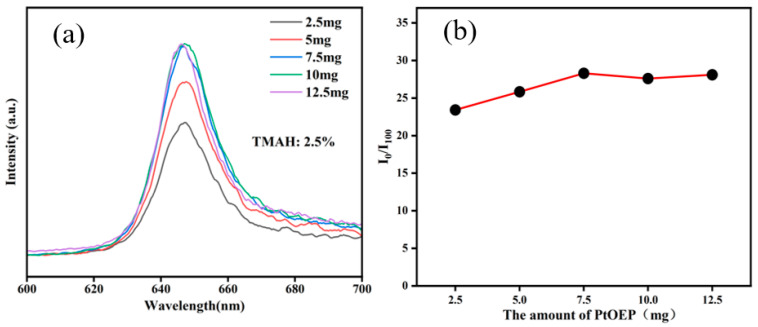
(**a**) Photoluminescence spectra and (**b**) quenching ratio (I_0_/I_100_) of PtOEP/SiO_2_ particles with different PtOEP additions.

With the optimal TMAH mass fraction fixed at 2.5 wt.%, PtOEP@SiO_2_ particles were synthesized using varying PtOEP amounts (2.5, 5, 7.5, 10, and 12.5 mg), and their photoluminescence and oxygen sensitivity characteristics are recorded in Figure 4. The results demonstrate that as the PtOEP content increases, the embedded PtOEP concentration in the films rises gradually, leading to the enhancement of photoluminescence intensity and oxygen sensitivity. However, no significant change occurs at higher PtOEP concentrations beyond 7.5 mg. The maximum I_0_/I_100_ value is also achieved at 7.5 mg PtOEP, which might be due to the concentration quenching of photoluminescence particles. Therefore, the optimal fluorescence performance and oxygen response capability is achieved when synthesizing silica-encapsulated PtOEP particles with 7.5 mg PtOEP and a 2.5 wt.% TMAH mass fraction.

**Figure 5:**
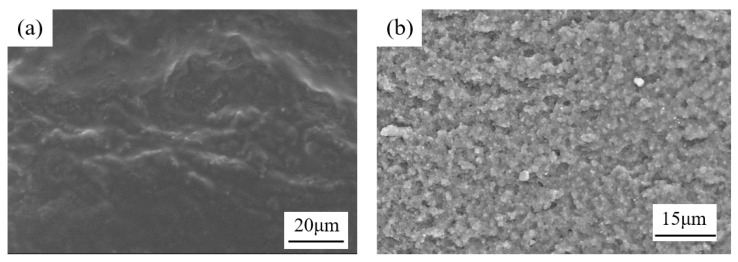
The SEM image of (**a**) the surface morphology and (**b**) the fracture structure of the as-prepared film.

Figure 5a shows the SEM image of the surface morphology of the as-prepared film, revealing numerous granular protrusions. This indicates that SiO_2_ particles are well-embedded within the silicone resin matrix. The uneven surface morphology further increases the contact area between the film and water. Figure 5b presents the fracture structure of the as-prepared film, demonstrating a highly porous structure. These pores facilitate oxygen diffusion within the film, enhancing the contact between oxygen molecules and particles, thereby improving the film’s oxygen response sensitivity. Figure 5a shows the SEM image of the film, revealing a porous internal structure. These pores enhance the contact between the DO in the water and the particles, thereby contributing to the high oxygen response capability of the film.

**Figure 6:**
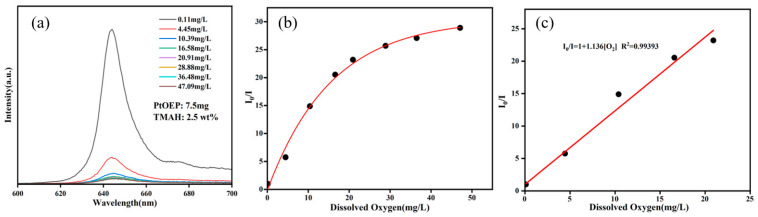
(**a**) Photoluminescence spectra of the film under different DO concentrations, (**b**) the Stern–Volmer plot at 0–47 mg/L DO concentrations, and (**c**) the Stern–Volmer plot at 0–20 mg/L DO concentrations.

To quantify oxygen sensitivity, DO concentration in deionized water was modulated by tailoring the container with O_2_ or N_2_. A commercial DO meter was used to measure the DO concentration; meanwhile, a photoluminescence spectrometer recorded the photoluminescence emission spectra. The photoluminescence intensity ratio was then utilized in the Stern–Volmer equation. Figure 6a displays the photoluminescence emission spectra excited at 380 nm under varying DO concentrations. Notably, the photoluminescence intensity decreases progressively as the DO concentration increases, demonstrating clear oxygen-dependent quenching. Figure 6b presents the Stern–Volmer calibration curve of the film over a DO range of 0~47 mg/L, revealing a nonlinear response to oxygen concentration. In contrast, Figure 6c focuses on the 0~20 mg/L DO range, where the film exhibits a strong linear response. The Stern–Volmer equation can then be shown as follows:

I_0_/I = 1 + 1.136[O_2_]  R^2^ = 0.99393 (1)

**Figure 7:**
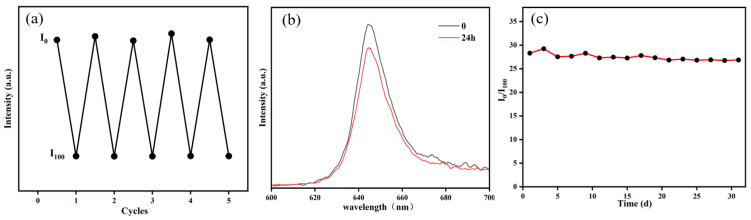
(**a**) Cyclic response diagram of the film’s I_0_ and I_100_, (**b**) emission spectra of the film before and after 24 h UV irradiation, and (**c**) effect of seawater immersion time on the film’s quenching ratio (I_0_/I_100_).

To establish zero-oxygen conditions, nitrogen gas was introduced into the container and the photoluminescence intensity (I_0_) was recorded. Subsequently, oxygen gas was supplied to create full-oxygen conditions and the photoluminescence intensity (I_100_) was measured. Five consecutive cycles were performed to evaluate the film’s reversibility. As shown in Figure 7a, no significant variation in I_0_ or I_100_ was observed after five cycles, confirming the film’s excellent reversibility. To evaluate the photostability of the thin film under prolonged excitation, the film was irradiated with UV light (20 μW/cm^2^) in a dark box for 24 h. Its emission spectrum was measured, as shown in Figure 7b. After 24 h of UV irradiation, the thin film still maintained 85.7% of its photoluminescence intensity, demonstrating excellent photostability. To test the stability of the thin film in seawater, seawater collected from the East China Sea with a salinity of 3.1% (data source: Bulletin of the East China Sea Branch of the Ministry of Natural Resources, China) was first subjected to centrifugation and filtration to remove microorganisms, thereby avoiding microbial adhesion that could affect the film’s photoluminescence intensity and oxygen response capability. Subsequently, 30 mL of the treated seawater was transferred into a flask, and the film was immersed in it. The sample was then stored under dark conditions at 25 °C. The I_0_/I_100_ ratio was measured every two days. After 30 days (Figure 7c), the I_0_/I_100_ ratio decreased by approximately 6%, demonstrating the film’s robust stability under marine conditions.

## 4. Conclusions

This study employed TMAH as a catalyst and successfully synthesized PtOEP@SiO_2_ nanoparticles with DO sensing capability via the Stöber method. The effects of TMAH concentration (1.0~3.0 wt.%) and PtOEP loading (2.5~10 mg) on particle size, photoluminescence intensity, and oxygen sensing performance were systematically investigated. Experimental results demonstrated that PtOEP@SiO_2_ nanoparticles prepared under optimized conditions (2.5 wt.% TMAH and 7.5 mg PtOEP) exhibited the best oxygen sensing performance. Subsequently, a film fabricated by drop-coating these nanoparticles embedded in a methyl silicone resin matrix achieved a quenching ratio (I_0_/I_100_) of 28 and a wide linear detection range (0~20 mg/L, R^2^ = 0.994). Stability tests revealed no significant performance degradation during five oxygen–nitrogen cycle tests. After 30 days of immersion in East China Sea seawater, the quenching ratio decreased by only 6%, confirming its excellent resistance to ion interference and long-term stability. This research provides a novel strategy for developing highly reliable in situ marine DO sensors.

## Data Availability

Data is contained within the article.
